# CovidGraph: a graph to fight COVID-19

**DOI:** 10.1093/bioinformatics/btac592

**Published:** 2022-08-30

**Authors:** Lea Gütebier, Tim Bleimehl, Ron Henkel, Jamie Munro, Sebastian Müller, Axel Morgner, Jakob Laenge, Anke Pachauer, Alexander Erdl, Jens Weimar, Kirsten Walther Langendorf, Vincent Vialard, Thorsten Liebig, Martin Preusse, Dagmar Waltemath, Alexander Jarasch

**Affiliations:** Medical Informatics Laboratory, University Medicine Greifswald, Greifswald 17475, Germany; HealthECCO, Freiburg 79098, Germany; HealthECCO, Freiburg 79098, Germany; German Center for Diabetes Research (DZD), Neuherberg 85764, Germany; Medical Informatics Laboratory, University Medicine Greifswald, Greifswald 17475, Germany; HealthECCO, Freiburg 79098, Germany; HealthECCO, Freiburg 79098, Germany; HealthECCO, Freiburg 79098, Germany; HealthECCO, Freiburg 79098, Germany; HealthECCO, Freiburg 79098, Germany; HealthECCO, Freiburg 79098, Germany; HealthECCO, Freiburg 79098, Germany; HealthECCO, Freiburg 79098, Germany; HealthECCO, Freiburg 79098, Germany; HealthECCO, Freiburg 79098, Germany; HealthECCO, Freiburg 79098, Germany; Medical Informatics Laboratory, University Medicine Greifswald, Greifswald 17475, Germany; HealthECCO, Freiburg 79098, Germany; German Center for Diabetes Research (DZD), Neuherberg 85764, Germany

## Abstract

**Summary:**

Reliable and integrated data are prerequisites for effective research on the recent coronavirus disease 2019 (COVID-19) pandemic. The CovidGraph project integrates and connects heterogeneous COVID-19 data in a knowledge graph, referred to as ‘CovidGraph’. It provides easy access to multiple data sources through a single point of entry and enables flexible data exploration.

**Availability and Implementation:**

More information on CovidGraph is available from the project website: https://healthecco.org/covidgraph/. Source code and documentation are provided on GitHub: https://github.com/covidgraph.

**Supplementary information:**

Supplementary data is available at *Bioinformatics* online.

## 1 Introduction

In 2019, a novel coronavirus emerged causing the worldwide coronavirus disease 2019 (COVID-19) pandemic. The SARS-CoV-2 virus, or severe acute respiratory syndrome coronavirus 2, infects humans and causes the life-threatening coronavirus disease. A lot of data, including biomedical research data, has become available since the outbreak. Collecting and connecting these data are essential in the understanding of the virus and the fight against the pandemic.

COVID-19-related data, e.g. genes, transcripts and proteins, quickly became available from different data domains. The data items are often described or used in publications, which themselves are accessible from different databases and repositories. In addition, patents, clinical trials and computational models predicting the spread of the disease have emerged. Consequently, researchers wishing to investigate COVID-19 causes, spread and related diseases need to know and consult multiple data domains. Due to heterogeneous data formats and a lack of common interfaces, this data collection process is time consuming and error prone. However, specifically during a pandemic, policymakers and health care providers need to act quickly to control the virus. Hence, a fast access, a correct integration and a central access point are needed.

The CovidGraph project implements these requirements and integrates COVID-19-related data into a knowledge graph, thus allowing for rapid and intuitive data exploration across different data domains. It is a project actively developed and maintained by HealthECCO (https://healthecco.org), an organization that focuses on data integration in the fields of medicine and biology. It was founded in 2020 and connects a team of professionals from research, data science and medicine who aim to help making data related to COVID-19 freely and easily accessible for researchers, health workers and policymakers in order to foster collaborative research.

## 2 CovidGraph

The CovidGraph project works towards creating a central hub of connected information serving as an overview and access point for important SARS-CoV-2 knowledge. As of November 2021, the knowledge graph contains publications, patents, clinical trials, biomedical data such as genes and other molecular data, statistical and geographical information, and systems biology data represented in a Neo4j graph database (https://neo4j.com/) with approximately 36 million nodes and 60 million relationships, continuously growing.

Information about *publications*, including authors and their affiliation, are integrated from the COVID-19 Open Research Dataset (CORD-19), a collection of COVID-19 and coronavirus-related research papers (Wang *et al.*, 2020). Information about *patents* related to COVID-19 is obtained from ‘The Lens’ (https://www.lens.org/). The public registry ClinicalTrials.gov (Zarin *et al.*, 2011) serves as the data domain for *clinical trials* that are related to COVID-19. *Biomedical entities* such as genes, transcripts and proteins are integrated from well-known genome databases, among others, Ensembl (Hubbard *et al.*, 2002), NCBI RefSeq (O’Leary *et al.*, 2016) and UniProt (The UniProt Consortium, 2021). Also included is information about pathways and gene expression data. Moreover, CovidGraph integrates functional annotation data from relevant ontologies, such as the Disease Ontology (Cowell and Smith, 2010). In addition, data are provided by the Hetionet resource (Himmelstein *et al.*, 2017), an integrative network of biomedical data. *Statistical data* such as case numbers is integrated from Johns Hopkins University (Dong *et al.*, 2020). The United Nations World Population Prospects 2019 (https://population.un.org/wpp/) offers population estimates and projections which are also integrated into CovidGraph. *Systems biology data*, in this case, simulation models and associated meta-data are integrated from MaSyMoS (Henkel *et al.*, 2015), which is a knowledge graph itself. MaSyMoS contains graph representations of models and meta-data from BioModels (Malik-Sheriff *et al.*, 2020), including a collection containing reproducible simulation studies of COVID-19 models (https://www.ebi.ac.uk/biomodels/covid-19).

A knowledge graph is most usable if, indeed, the knowledge is connected. Therefore, mappings across data domains are specified and these connections implemented in CovidGraph. One example of connected domains is literature and ontological knowledge. The corpus of included *publications*, *patents* and *clinical trials* is processed and occurrences of biomedical terms are recognized. Subsequently, recognized terms are mapped to their corresponding biomedical ontology entries (Gütebier *et al.*, 2021).

## 3 Interfaces and availability

CovidGraph enables easy browsing across different data sources. Data exploration can be started via one of four user interfaces or programmatically via an API. Each interface is tailored towards a specific exploration approach. The interfaces as described below can be accessed through the CovidGraph website (https://healthecco.org/covidgraph/).

The *Visual Graph Explorer* by yWorks provides a variety of predefined views for an intuitive keyword-based graph exploration (Supplementary Fig. S2). No prior knowledge of database query languages or the underlying graph structure is necessary for this straightforward approach. Users can interact with the clear interface by entering keywords in the search bar and filtering them for entities such as papers, patents, genes and more. Results are displayed as easily understandable connected glyphs allowing for a customized view of the selected data. When selecting a glyph (e.g. the gene *ACE2*), the user can load all related information (e.g. all encoded proteins). On request, additional information about the returned glyphs is provided in the detail panel. Moreover, the user can retrieve the underlying database queries for additional exploration options.

*SemSpect* (Liebig *et al.*, 2017) provided by derive (https://www.derivo.de/) offers unfiltered access to the database via an interactive drag-and-drop exploration tool (Supplementary Fig. S1). Data items selected by the user are automatically aggregated in groups (e.g. synonyms for the gene *ACE2*), and relations between different groups are displayed in an expandable tree structure. The user can filter, select and highlight single nodes inside a group (e.g. the gene *ACE2*), thereby building a tailored visual representation of the data without detailed knowledge of the underlying graph structure or query language. The resulting, customized visualization can be exported.

Experienced users may access the database directly through Cypher queries and in combination with visual exploration, offering more sophisticated access to the data. The interface is provided by the build-in *Neo4j Browser*.

More precisely, the integrated command line facilitates pattern matching on the graph database by entering specific queries for pattern and paths. Query results are returned as a visual representation of the resulting graph or, depending on the user request, as a tabular format or attribute-value pairs. The interface also includes the Graph Data Science and Awesome Procedures On Cypher libraries (https://neo4j.com/developer/graph-platform/). Both allow the user to natively use common graph algorithms (e.g. Dijkstra) or a variety of data import, export and manipulation methods, respectively.

**Fig. 1.**
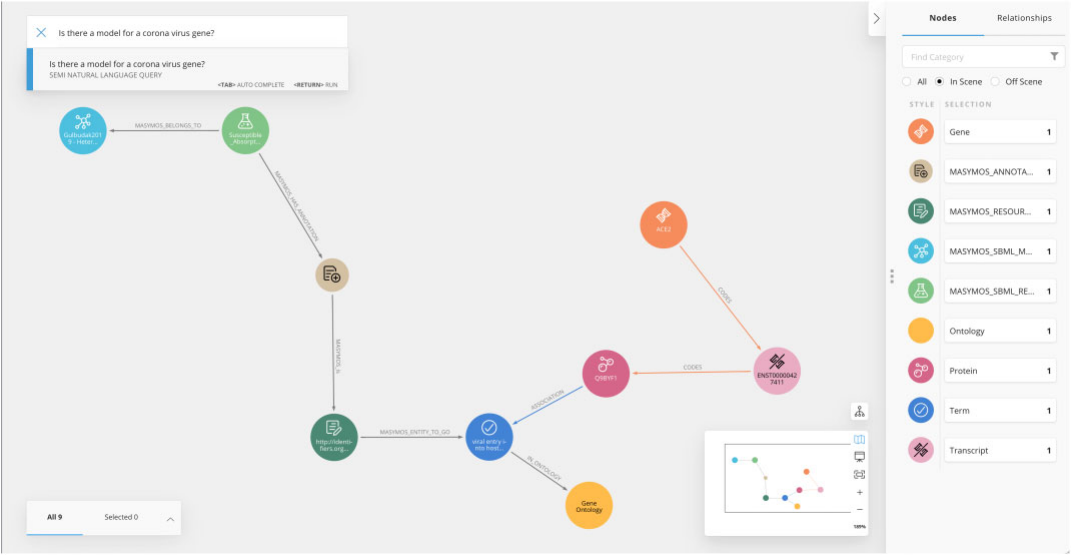
Screenshot of Neo4j Bloom with semi-natural language query ‘Is there a model for a corona virus gene?’. This figure shows the shortest path between the systems biology model ‘Gulbudak2019.1—Heterogeneous viral strategies promote coexistence in virus-microbe systems (Lytic)’ (cyan, BioModels, BIOMD0000000845) by Gulbudak and Weitz (2019) which is connected to MaSyMoS_Reaction (light green), MaSyMoS_Annotation (ochre), MaSyMoS_Resource (green) to the Gene Ontology term ‘Virus entry into host cell’ (blue, GO: 0019063) which, in turn, is associated with the protein Q9BYF1 (plum, UniProt) coded by the transcript ENST00000427411 (pink, RefSeq) of the corona virus gene *ACE2* (orange, GenBank, ENSG00000130234) (A color version of this figure appears in the online version of this article.)

*Neo4j Bloom* is an easy-to-use graph exploration application for visually interacting with Neo4j databases (Fig. 1). Neo4j Bloom provides easy-to-configure graph visualization, as well as rule-based styling for nodes or relationships. Using semi-natural language queries (https://neo4j.com/product/bloom/) allows non-computer scientists researchers to easily query the graph databases by typing phrases and sentences in the search bar.

In addition, the Neo4j API is available for a programmatic access. It enables connection with external tools and programs for automatic querying or downloading of the data.

## 4 Use case

The cell surface receptor that is utilized by SARS-CoV-2 to enter the host cells is called angiotensin-converting enzyme 2 (*ACE2*). During the cell entry, the receptor acts as a binding site for the spike protein of the coronavirus Ni *et al.* (2020). To gain more knowledge and a better understanding about the mechanisms involved in a coronavirus infection, we want to investigate the underlying biological processes. CovidGraph connects important knowledge about the *ACE2* receptor with existing simulation models. Simulation models can assist in research by describing a biological system in a machine-readable format and enabling computational simulation and analysis of the system.

For our use case, we run the predefined query ‘Are there systems biology models for the gene *ACE2*?’ in Bloom which returns a set of fitting simulation models within seconds. Figure 1 shows one of the identified models and the connecting path between the data domains. Next, we identify suitable models within the resulting set to investigate the above-mentioned mechanisms underlying a coronavirus infection. Therefore, we again query the data in CovidGraph. The query ‘Which models are related to coronavirus entry into host cell?’ makes use of the ontological term ‘viral entry into host cell’ represented in a node and returns relevant models. The returned models include three models on HIV infection, a virus that enters the host cell via membrane fusion, and a model on influenza virus replication describing a receptor-mediated endocytosis (Heldt *et al.*, 2012). Membrane fusion and endocytosis are two main mechanisms for viral entry into cells (Jackson *et al.*, 2022). Based on previous knowledge, these models can help to gain insights into the entry mechanisms of the coronavirus.

The use of open data and open software tools, including easy access and exploration of the integrated, heterogeneous COVID-19 data, makes the CovidGraph a FAIR resource (Wilkinson *et al.*, 2016). The user is provided with a *F*indable, *A*ccessible, *I*nteroperable, *R*euseable resource that can be easily and intuitively explored with the aforementioned interfaces. CovidGraph’s open-source policy and free accessibility allow for an unrestricted usage and, in fact, for integration of further data sources. Its modular framework encourages the addition of new data sources and supports periodical updates of the integrated data, an update strategy we are currently implementing.

## Supplementary Material

btac592_Supplementary_Data
