# Supplementary material for: CovidGraph: a graph to fight COVID-19
Source: Bioinformatics. 2022 Aug 30;38(20):4843–5. doi: 10.1093/bioinformatics/btac592 (PMC9563682; doi:10.1093/bioinformatics/btac592)
Supplement: btac592_Supplementary_Data [file btac592_supplementary_data.pdf]

Supplementary material

The HealthECCO website is located at `https://healthecco.org/`.
The CovidGraph project is part of HealthECCO. For the website of the project see `https://healthecco.org/covidgraph/`.

1 List of Data Sources

CORD-19 The COVID-19 Open Research Dataset (CORD-19) from the CORD-19 Research Challenge is a collection of publications related to COVID-19 and the coronavirus family.

`https://www.semanticscholar.org/cord19`, `https://www.kaggle.com/allen-institute-for-ai/CORD-19-research-challenge`

The Lens The Lens offers datasets of COVID-19 patents.

`https://about.lens.org/covid-19/`

ClinicalTrials.gov The ClinicalTrials.gov resource provides clinical trial data, including clinical trials concerning COVID-19.

`https://clinicaltrials.gov/`

Ensembl Genome Browser Ensembl is a genome database that includes information about genes.

`http://www.ensembl.org/index.html`

NCBI Gene The NCBI Gene Database provides information about genes.

`https://www.ncbi.nlm.nih.gov/gene/`

NCBI RefSeq The NCBI Reference Sequence Database is a collection of reference sequences including transcripts.

`https://www.ncbi.nlm.nih.gov/refseq/`

UniProt The Universal Protein Resource contains protein sequences and associated functional data.

`https://www.uniprot.org/`

Reactome Reactome is a pathway database that stores information about biological pathways.

`https://reactome.org/`

GTEx Portal The GTEx Portal is a tissue database containing gene expression data.

https://gtexportal.org/home/

Disease Ontology The Infectious Disease Ontology is an ontology for human diseases.

https://disease-ontology.org/

Gene Ontology The Gene Ontology resource comprises information about genes, their functions and products.

http://geneontology.org/

Human Phenotype Ontology Terms in the Human Phenotype Ontology represent phenotypes of human hereditary diseases.

https://hpo.jax.org/app/

Phenotype and Trait Ontology The Phenotype and Trait Ontology contains information about phenotypic qualities.

http://www.obofoundry.org/ontology/pato.html

Mammalian Phenotype Ontology The Mammalian Phenotype Ontology describes mammalian phenotypes.

http://www.informatics.jax.org/vocab/mp_ontology/

ChEBI ChEBI, short for Chemical Entities of Biological Interest, is an ontology for molecular entities such as metabolites.

https://www.ebi.ac.uk/chebi/

Hetionet The biomedical data network Hetionet includes information about diseases and anatomy.

https://het.io/

Johns Hopkins University The Johns Hopkins University Center for Systems Science and Engineering (JHU CSSE) developed the 2019 Novel Coronavirus COVID-19 (2019-nCoV) Data Repository, a data repository for the COVID-19 Dashboard.

https://coronavirus.jhu.edu/map.html

United Nations World Population Prospects 2019 The World Population Prospects include official population estimates and projections from the United Nations.

https://population.un.org/wpp/

MaSyMoS The Management System for Models and Simulations (MaSyMoS) contains graph representations of simulation models and associated metadata. The original models are hosted at BioModels.

https://masymos.readthedocs.io, https://www.ebi.ac.uk/biomodels/

As of now[1], integrated data in CovidGraph from these data sources includes 128053 publications, 32080 patents, 6185 clinical trials, 895539 genes, 1270615 transcripts and 1058594 proteins, 20567 pathways, 22951 GTEx samples, 13177 Disease Ontology terms, 47210 Gene Ontology terms, 16116 Human Phenotype Ontology terms, 2687 Phenotype and Trait Ontology terms, 13698 Mammalian Phenotype Ontology terms, 137654 ChEBI entries and 1002 simulation models.

2 Interfaces and Videos

The *Visual Graph Explorer* by yWorks (`https://www.yworks.com/`) provides a variety of predefined views for an intuitive keyword-based graph exploration. It can be accessed at `https://live.yworks.com/covidgraph/`. No login is required.
SemSpect provided by derivo (`https://www.derivo.de/`) offers unfiltered access to the database via an interactive drag-and-drop exploration tool. To explore CovidGraph with SemSpect, users can log in at `https://db.covidgraph.org/semspect/` (Username: public, Password: corona).
Experienced users may access the database directly through Cypher queries and in combination with visual exploration. The interface is provided by the build-in *Neo4j Browser* at `https://db.covidgraph.org/browser/` (Username: public, Password: corona). The Neo4j Browser interface includes the Graph Data Science (GDS) and Awesome Procedures On Cypher (APOC) libraries. For further information about the GDS library see `https://neo4j.com/product/graph-data-science-library/` and for details about the APOC library see `https://neo4j.com/developer/neo4j-apoc/`.
Neo4j Bloom is an easy-to-use graph exploration application for visually interacting with Neo4j databases. The Neo4j Bloom interface for CovidGraph can be accessed at `https://db.covidgraph.org/browser/bloom/` (Username: public, Password: corona). Phrases and sentences can be typed directly in the search bar by using semi-natural language queries (`https://neo4j.com/product/bloom/`).

HealthECCO runs a YouTube channel (`https://www.youtube.com/channel/UCkHF_MYZXwPSl6OPVs4sxEQ`) with example uses of the various interfaces and presentations about CovidGraph.
The following video descriptions relate to the figures in the application note (see Section 5 in supplementary material). Links to access the corresponding videos are given below.

Visual Graph Explorer The video shows an example for the use of the Visual Graph Explorer. The interface includes a search bar for entering keywords, a filter for selecting entities, a detail panel and a screen displaying the loaded data. After loading the gene ACE2 related information can be accessed. Therefore, a click on the gene loads pathways in which ACE2 is

[1] November 2021

active, proteins that are encoded by the gene, and tissues in which ACE2 is expressed. In addition, mentioning papers, related authors and affiliations are loaded. Information about related patents is also displayed.

https://www.youtube.com/watch?v=Qn2jvHS7mcE

SemSpect This video explains how to explore CovidGraph with SemSpect. For the exploration, the label Gene is selected from a list of node labels and dragged to the blank screen of the interface, thereby creating a group of nodes on the screen. By opening the node table of a selected group and filtering for keywords, the user can restrict the displayed nodes inside the corresponding group. In the video, the exploration is focused on the gene PF4 and its synonyms, respectively. It is shown, how patents and papers are connected to the genes by text-fragments and how the underlying biological connections can be explored.

https://www.youtube.com/watch?v=AGDNExH2B0I

Neo4j Bloom This video shows how to use the Neo4j Bloom interface of CovidGraph for semi-natural language queries. Therefore, the example query "Is there a model for a corona virus gene?" is entered into the search bar of the interface. A graph displaying the shortest path between a model and a gene is returned. Corresponding nodes labels are represented by coloured symbols.

https://www.youtube.com/watch?v=mc8QPV15C_8

3 GitHub organisation

Source code and documentation are provided on GitHub at https://github.com/covidgraph. The documentation repository (https://github.com/covidgraph/documentation) provides information about CovidGraph and contains the main wiki page. CovidGraph relies on a data loading pipeline and data loading scripts. The data loading scripts transform the source data into a graph structure before loading the data into the database. The repositories that are needed for setting up CovidGraph are organised as follows:

motherlode Motherlode is the pipeline for running all data loading scripts for CovidGraph in a controlled manner. The motherlode repository includes instructions on how to run the pipeline.

https://github.com/covidgraph/motherlode

data_template The data_template repository contains a template for writing a data loading script for CovidGraph in Python.

https://github.com/covidgraph/data_template

graph-processing_fulltext-indexes This repository contains a script that creates full-text indexes in CovidGraph.

https://github.com/covidgraph/graph-processing_fulltext-indexes

data_jhu_population The data_jhu_population repository provides scripts for loading COVID-19 population data and case statistics into CovidGraph.

https://github.com/covidgraph/data_jhu_population

data-lens-org-covid19-patents This repository contains a script for loading COVID-19 patent data into CovidGraph.

https://github.com/covidgraph/data-lens-org-covid19-patents

data_cord19 The script in this repository loads the CORD-19 dataset into CovidGraph.

https://github.com/covidgraph/data_cord19

graph-processing_fragmentize_text This repository contains code to split text from full-text data such as publications and patents into fragments and to create fragment nodes in CovidGraph.

https://github.com/covidgraph/graph-processing_fragmentize_text

graph-processing_text_gene_match The code in this repository matches the created fragments with genes that are mentioned in these fragments and creates relationships between the corresponding nodes.

https://github.com/covidgraph/graph-processing_text_gene_match

data_clinical-trials-gov The data loading script in this repository integrates clinical trial data related to COVID-19 into CovidGraph.

https://github.com/covidgraph/data_clinical-trials-gov

covidgraph-data-hetionet The repository loads data from Hetionet into the graph database.

https://github.com/covidgraph/covidgraph-data-hetionet

data_biobert The data_biobert repository contains pre-processed data from BioBERT and code for named entity recognition.

https://github.com/covidgraph/data_biobert

data_MaSyMos The data_MaSyMos repository contains the data loading script for systems biology models and associated meta-data.

https://github.com/covidgraph/data_MaSyMos

4 Server details

CovidGraph has a production (publicly available) instance. The instance has a Neo4j Enterprise version 4.3.5. running. The database of the production instance currently contains 21 GB data, reflecting the aforementioned data sources represented as a graph. For further details about the server and the server specification see `https://github.com/covidgraph/documentation/wiki/Production-Server`.

5 Maintainability and Update strategy

The database is maintained by the HealthECCO organisation aiming to provide the data in an easily accessible fashion. Although we recognise the importance of keeping the data up-to-date, regular updates are not yet implemented. Nevertheless, the sophisticated design of the database framework allows for straightforward modification to implement updates. In fact, we are already working on implementing an update strategy of CovidGraph. A next iteration of the knowledge graph is planned and will include a weekly update of the data.

6 Performance

Our goal is to provide queries that return results within reasonable time, optimally within seconds.
A graph database is designed to efficiently process path queries. However, complex graph queries may reach hundreds of nodes can take several minutes - entirely depending on the query itself.
Due to certain limitations, including limitations on client side and finite server resources, we cannot guarantee a timely or successful execution of each user query. The database engine is configured to limit query outputs to a certain number of nodes and also limits a queries execution time in order to keep the system as a whole stable for all users. This may result in queries to timeout or fail. Same holds for the different interfaces - as, for example, Neo4j is not able to visualise thousands of nodes as a query result - also taking into account that such an output is not beneficial for a user. Thus, a general statement about performance of the interfaces and expected query times is difficult to formulate. A rule of thumb which a user can rely on is that the performance of a query generally depends on the number of involved nodes and their connections in the graph.
In Bloom, the predefined queries are designed to return results in reasonable time.

7 Figures

The following figures show the interface of SemSpect (Fig. 1) and a screenshot of the Visual Graph Explorer (Fig. 2).

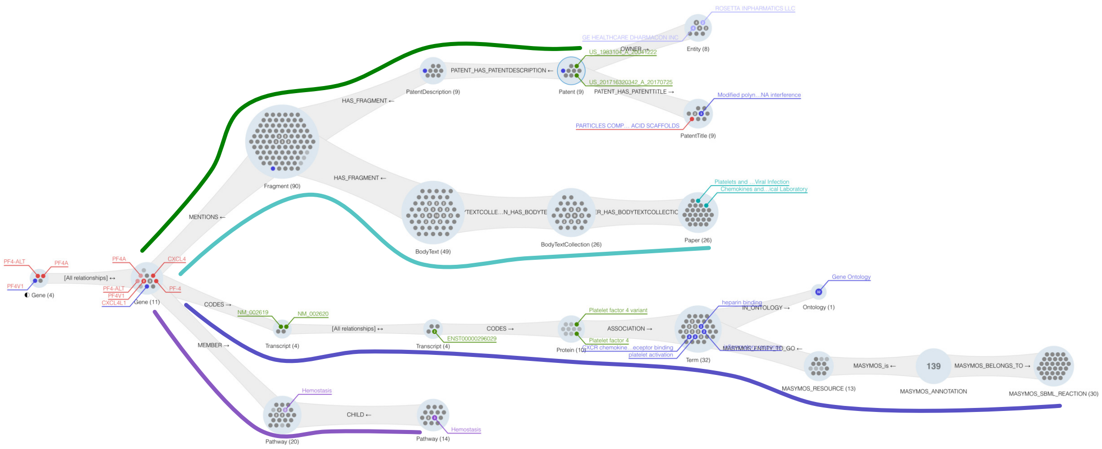

Figure 1: SemSpect tree representation (the coloured lines are not part of the SemSpect rendering). The figure displays a tree structure starting with the gene PF4 (on the left) and its synonyms. Related patents, publications, proteins, models and pathways are show in different tree branches. Synonyms of the gene PF4 are mentioned in text fragments. These fragments are part of a patent description (see green line), or belong to a text written in a paper (see cyan line). The longest branch crosses multiple data domains, including biological entities, ontology terms, and simulation models (see blue line). A hierarchy of pathways containing the gene PF4 or its synonyms can be seen in the lowest branch (see purple line). Rather than showing relationships between nodes within a group, the interface displays relationships across different domains and aggregates them according to their type. Selected nodes are highlighted with a tag, e.g. displaying gene names.

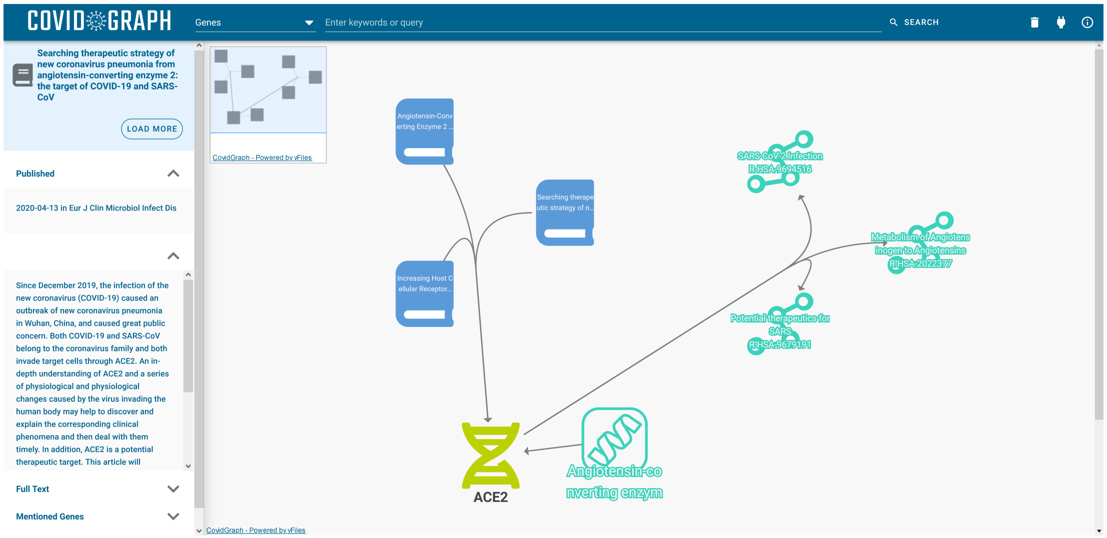

Figure 2: Screenshot of the Visual Graph Explorer. The figure displays a diagram of the COVID-related gene ACE2 with encoded proteins, related publications and pathways as connected glyphs. Each entity is represented by a glyph, e.g. the entity gene is pictured as a DNA symbol. Arrows between glyphs indicate an underlying relationship between the entities. The detail panel on the left includes information about a selected publication. Keywords can be conveniently entered in the search bar on the top and filtered for various entities.
